# Supplementary material for: Modifying the Secretome of Mesenchymal Stem Cells Prolongs the Regenerative Treatment Window for Encephalopathy of Prematurity
Source: Int J Mol Sci. 2024 Jun 12;25(12):6494. doi: 10.3390/ijms25126494 (PMC11203777; doi:10.3390/ijms25126494)
Supplement: Supplementary file 1 [file ijms-25-06494-s001.zip › ijms-2897094-supplementary.pdf]

Supplementary Materials

Modifying the secretome of MSCs prolongs the regenerative treatment window for Encephalopathy of Prematurity

Josine E.G. Vaes[1,2], Suzanne M. Onstwedder[1,†], Chloe Trayford[3,,†], Eva Gubbins[3], Mirjam Maas[1], Sabine H. van Rijt[3], Cora H. Nijboer[1,*]

[1] Department for Developmental Origins of Disease, University Medical Center Utrecht Brain Center and Wilhelmina Children's Hospital, Utrecht University, Utrecht, The Netherlands
[2] Department of Neonatology, University Medical Center Utrecht Brain Center and Wilhelmina Children's Hospital, Utrecht University, Utrecht, The Netherlands
[3] Department of Instructive Biomaterials Engineering, MERLN Institute for Technology-Inspired Regenerative Medicine, Maastricht University, Maastricht, The Netherlands
* Correspondence: C.Nijboer@umcutrecht.nl; Tel.: +31 88 755 4360
† These authors contributed equally to this work and share the second authorship.

This file includes:

- Supporting text Materials and Methods
- Table S1
- Table S2
- Table S3
- Table S4
- Figure S1

Supporting Materials and Methods

MSC culture

GIBCO® mouse (C57BL/6) bone marrow-derived MSCs (Invitrogen, S1502-100; Carlsbad, California, USA) were cultured in D-MEM/F-12 medium (10565-018, Invitrogen) with 10% fetal bovine serum (12662-029, Invitrogen) according to the supplier's protocol. In all experiments, MSCs were passaged once (from P2 to P3) prior to *in vivo* administration or in *in vitro* experiments.

MSC transfection

MSCs were modified to transiently overexpress growth factor or cytokines with ready-to-use recombinant adenoviral vectors encoding a murine IGF1, EGF, LIF, IL11 or IL10 transgene, combined with a control eGFP vector to assess infection efficacy (Vector Biolabs, Malvern, US). Transgenes were placed under control of a CMV promotor, including a separate promotor for eGFP. To stimulate cell entry, an Arg-Gly-Asp (RGD) motif was included, due to poor basal expression of the coxsackievirus-adenovirus receptor (CAR) in MSCs. 24 hours prior to viral particle exposure, MSCs were plated at 2.0×10^5 cells per well in 6-wells plates to allow cell adherence. The following day, MSCs were exposed to viral particles diluted in MSC culture medium for 6 hours. Subsequently, wells were washed with culture medium and recultured for 24 hours followed by *in vitro* gel embedment or *in vivo* administration. Transfection was visually confirmed by evaluation of the eGFP signal. The optimal

multiplicity of infection (viral particles needed per cell; MOI) was determined per adenovirus, by assessment of secreted protein concentrations by MSCs in supernatants using ELISA (at 2 days after infection). All ELISAs were performed according to manufacturer's protocol (mouse IGF1: MG100, R&D systems; mouse EGF: EMEGF, Invitrogen; mouse LIF: ABIN5526767, antibodies online; mouse IL11: RAB0251, Sigma; mouse IL10: ABIN2114255, antibodies online).

***In vivo* model of diffuse white matter injury**

All procedures were carried out according to the Dutch and European guidelines (Directive 86/609, ETS 123, Annex II) and were approved by the Experimental Animal Committee Utrecht (Utrecht University, Utrecht, Netherlands) and the Central Authority for Scientific Procedures on Animals (the Hague, the Netherlands). Diffuse white matter injury was induced as described previously (1). In short, hypoxia-ischemia (HI) was induced in postnatal day 5 (P5) C57BL/6j mouse pups by permanent unilateral occlusion of the right common carotid artery and exposure to hypoxia (6% O_2) for 35 minutes under temperature-controlled conditions. Directly after hypoxia, pups received an intraperitoneal (i.p.) injection with 1mg/kg LPS (List Biological Laboratories, Campbell, CA) dissolved in 0.9% NaCl. Sham-control littermates underwent surgical incision only, without carotid artery occlusion, nor hypoxia or LPS injection. Prior to MSC administration at P8 (i.e. D3 after dWMI induction) or P11 (i.e. D6) nasal mucosa permeability was improved by administration of 2 dosages of 2µl Hyaluronidase (12.5 U/µl in total, Sigma-Aldrich, St. louis, MO) dissolved in H_2O in each nostril (total of 8 µl). Thirty minutes after hyaluronidase treatment, $0.5x10^6$ MSCs were administered intranasally in dPBS (Thermo-fisher, 14190-169, Waltham, MA) in 2 dosages of 2µl in each nostril (total of 8µl). Previous dose-response experiments identified $0.5x10^6$ MSCs as the lowest effective dose (1). Vehicle-treated dWMI animals received 8µl dPBS (2 dosages of 2µl in each nostril). Mice were euthanized at P8 (i.e. 3 days), P11 (i.e. 6 days) or P26 (i.e. 3 weeks) by an i.p. overdose pentobarbital. For PCR arrays sham-control or dWMI (untreated) brains were collected, cerebellum was discarded and hemispheres were separately snap-frozen in liquid nitrogen and stored at -80°C until further processing.

Cerebral chemokine expression profiles

For cerebral chemokine expression analysis sham-control and dWMI (untreated) brains were collected at two time points after dWMI induction, P8 (i.e. 3 days) and P11 (i.e. 6 days). The ipsilateral hemisphere of each brain was crushed using a mortar and pestle chilled on liquid nitrogen. Brain tissue was lysated in RLT lysis buffer using a TissueLyser LT Adapter and stainless-steel beads (all Qiagen, Hilden, DE), at 50Hz during 2 minutes according to manufacturer's protocol. RNA was isolated using the RNeasy minikit (Qiagen), including on-column DNase digestion with the RNase-free DNase set (79254, Qiagen). RNA quantity and quality were assessed by spectrophotometry (NanoDrop 2000, Thermo-scientific, Waltham, MS) at 260mm and OD 260/280 ratio. cDNA transcription was carried out using the RT2 first strand kit (Qiagen), following manufacturer's protocol. cDNA of sham-control or dWMI animals were pooled per time point (D3 n=5 and D6 n=4 per experimental condition) and the expression of 84 chemokine and cytokine-related genes were assessed in a commercially-available PCR array (PAMM-150Z, Qiagen). PCR arrays were carried out following suppliers' protocol, using the RT2 Real-Time SYBR green PCR Master Mix (Qiagen) on a Biorad MyIQ. PCR array data were normalized using multiple housekeeping genes incorporated per array, and analyzed by comparing 2-ΔCt using software provided by Qiagen. Chemokine gene expression changes were calculated: 1) in dWMI mice relative to sham-control mice at P8 (i.e. 3 days) to identify chemokines that are differentially regulated following injury induction and 2) in dWMI mice at P11 (i.e. 6 days) versus dWMI mice at P8 (i.e. 3 days) to study the stability of the chemotactic signals over time. A fold regulation threshold of 3.0 was

considered as either down- or upregulation. PCR array results were validated by quantitative PCR analyses in the individual cDNA samples for selected genes (CXL10 and Ccl3). Real time RT-PCR was carried out using the QuantStudio 3 (Applied Biosystems) with SYBR select master mix (Applied Biosystems). Primer sequences can be found in table S1. Mean expression of GADPH and β-actin were used for data normalization.

MSC gene expression profiles after exposure to brain extracts

To evaluate the response of the MSC secretome to the cerebral milieu at two time points after dWMI induction, dWMI brains were collected at D3 (n=5) and D6 (n=4). The ipsilateral hemispheres were crushed on liquid nitrogen using a mortar and pestle. After weighing of the tissue pieces, tissue of both time points was pooled and homogenized at 150 mg/ml in knock-out DMEM (Thermo Fisher, 10829018) containing a protease inhibitor cocktail (1:50 dilution; Invitrogen) using a potter tissue homogenizer (10 strokes), followed by centrifugation for 10 minutes at 10,000 x g at 4°C. The supernatant 'brain extract' was collected and the protein concentration assessed using a protein assay (Biorad) with BSA as standard. Brain extracts were aliquoted and kept at -80C until use. MSCs were cultured and seeded at $2.0x10^5$ cells per well (6-wells plate) in standard MSC medium. After allowing the cells to adhere during 24 hours, culture medium was replaced with knock-out DMEM containing either D3 or D6 dWMI brain extract at a concentration of 1mg protein/ml. Wells were washed with ice-cold PBS 48 hours after addition of the brain extract-enriched medium. MSC RNA was isolated using the RNeasy minikit (Qiagen). RNA quantity and quality were assessed by spectrophotometry (NanoDrop 2000, Thermo-scientific, Waltham, MS) at 260mm and OD 260/280 ratio was determined to evaluate quality. Transcription of RNA to cDNA was performed using the RT2 first strand synthesis kit (Qiagen) according to manufacturer's instructions. The expression profiles of 168 growth factor- and cytokine-related genes were assessed by commercially available PCR arrays (Qiagen; PAMM-041Z and PAMM-150Z). PCR array analysis was performed once following manufacturer's protocol with the RT2 Real-Time SYBR green PCR Master Mix (Qiagen) on QuantStudio 3 (Applied Biosystems). PCR data were normalized using multiple housekeeping genes provided within the PCR array and analyzed by comparing 2-ΔCt using Qiagen software. Gene expression changes in MSCs exposed to D6 dWMI brain extracts were calculated relative to MSCs exposed to brain extracts obtained at D3 after injury induction. A fold regulation threshold of 3.0 was considered as either down- or upregulation. As described in the previous section, PCR arrays results were validated by quantitative PCR analyses in the individual cDNA samples for selected genes (IL1β and Ccl3). Primer sequences can be found in table S1. Mean expression of GADPH and β-actin were used for data normalization.

MSC tracing

<u>MSC labeling</u>

MSCs were labelled using gold core-mesoporous and lipid-coated silica nanoparticles (AuNP-MSN-LIP). A detailed description of nanoparticle synthesis, characterization and labeling efficiency can be found in our previous paper (1). In short, 2 hours after cell passaging, MSCs were incubated with 25μg/ml AuNP-MSN-LIP in culture medium over 48 hours. Following cell labeling, dWMI animals received intranasal treatment with $0.5x10^6$ MSCs in PBS at P8 or at P11.

<u>Cell tracing in mouse tissue</u>

Mice were sacrificed by overdose pentobarbital following by decapitation at 12 hours after intranasal MSC treatment. The brains were collected, divided in ipsilateral and contralateral hemispheres, cerebellum and olfactory bulbs and frozen separately in liquid nitrogen. Additionally, the spleens, lungs and livers were dissected and frozen in liquid nitrogen to study loss of cells in peripheral organs. Inductively coupled plasma mass spectrometry (ICP-MS) was used to quantitatively assess MSC biodistribution by detection of gold in mouse tissue homogenates, as described previously (1). In summary, snap frozen tissue sections were weighed, lyophilized overnight and reweighed. Freshly prepared aqua regia (HCl 30% and HNO3 60%, VWR) was added to each tissue section for w/v; 1mg/50µL. Samples were disintegrated overnight at 40°C using an ultrasonic bath (Bransonic®, Thermo Scientific) and further homogenized by microwaving (5x30 seconds, 600W). When solutions were transparent, all tissue samples were diluted 1:10 in freshly prepared matrix solution (1% HNO3 functionalized with 20ppb of ruthenium (VWR)). In addition, a gold standard curve ranging from 1ng L-1 to 100µg L-1 was made by diluting gold stock solution (VWR) in the prepared matrix. An iCAP™ RQ ICP-MS (Thermo Scientific) was used to measure ICP-MS. Due to its high molecular weight of 197 other ions will not interfere with measured gold content. The detection limit of gold was 1ng/L.

Immunohistochemistry

Animals were sacrificed by overdose pentobarbital followed by transcardial perfusion with PBS and 4% PFA in PBS at P26 (i.e. 3 weeks). Brains were post-fixed during 24 hours in 4% PFA followed by a dehydration series. Brains were then embedded in paraffin and coronal sections (8μm) were cut at hippocampal level (-1.80mm from bregma in adult mice). For 3,3'-Diaminobenzidine (DAB) staining, sections were deparaffinized in xylene followed by 100% ethanol. Endogenous peroxidase was blocked by incubation in 3% H2O2/methanol and sections were hydrated using decreasing concentrations of ethanol. Sections were blocked with 20% normal rabbit serum in PBS/0.1% Tween followed by overnight incubation with rat-anti-MBP (MAB386, Merck Millipore; 1:500) in 10% normal rabbit serum in PBS/0.1% Tween. The next day, sections were washed in PBS and incubated with biotinylated rabbit-anti-rat (BA-4000, Vector laboratories, 1:400), followed by PBS washes. Biotin was HRP-labeled using a vectastain ABC kit (Vector laboratories) according to the supplier's protocol, followed by 0.05M Tris-HCl (pH: 7.6) washing. Subsequently, sections were stained with 0.5mg/ml DAB (Sigma) in 0.05M Tris-HCl with 0.03% H_2O_2. Sections were washed in H2O, dehydrated in increasing ethanol concentrations and embedded with depex. For immunofluorescent stainings, sections were deparaffinized in xylene, followed by rehydration in decreasing concentrations of ethanol. Sections were heated to 95°C in sodium citrate buffer (0.01M, pH 6) for antigen retrieval. After cooling down and washing in PBS (+0.1%Tween20 for MBP/NF200), sections were blocked with 10% normal goat serum in PBS+0.1% Tween20 for MBP/NF200 or 2% bovine serum albumin (BSA)/0.1% saponin in PBS for Iba1 staining and incubated overnight with rat-anti-MBP (MAB386, Merck Millipore; 1:500), rabbit-anti-NF200 (N-4142, Sigma; 1:400), rabbit-anti-Iba1 (019-19741, Wako; 1:500) and with mouse-anti-GFAP (BM2278, Origine; 1:200). The next day, sections were washed in PBS and incubated with alexafluor-594 and -488 conjugated secondary antibodies (Life technologies, Carlsbad, CA; 1:200-500) for 1-2 hours at room temperature, followed by DAPI (1:5000) counterstaining and embedment in Fluorsave (Merck Millipore, 345789).

Microscopy and image analysis

Investigators were blinded for experimental conditions during image acquisition and analysis. In MBP-DAB-stained sections a 2.5x magnification was used to image the ipsilateral hemisphere using a light microscope (Zeiss, Oberkochen, Germany) with an AxioCam ICc 5 camera (Zeiss). For immunofluorescent stainings, a Cell Observer microscope with an AxioCam MRm camera (Zeiss, Oberkochen, Germany) was used to acquire images in the ipsilateral hemisphere. For MBP/NF200 stainings, 3 adjacent 40x micrographs were taken at a fixed distance of the external capsule into the cortex (for exact locations we refer to (2)). For Iba1 and GFAP stainings, two 20x images were acquired in the corpus callosum of the ipsilateral hemisphere. In addition, one 20x image of the CA1 region of the hippocampus (dorsal from the dentate gyrus) was obtained for GFAP area analyses. In MBP-DAB stainings, cortical myelination (2.5x) was quantified as described by van Tilborg, van Kammen (2). In addition, microstructural integrity of myelinated axons (40x) was analyzed in MBP/NF200 stained sections as described in van Tilborg, van Kammen (2). Morphology of microglia residing in the corpus callosum was assessed using the particle analysis function of ImageJ v.1.47 (3) as described by (4). In these analyses, microglia were manually selected followed by morphological skeleton measurements. GFAP threshold analyses to calculate the positive area of staining, were carried out using ImageJ software (v.1.47). Values of all acquired images were averaged per animal.

Behavioral assessment

Motor performance was evaluated using the cylinder rearing test (CRT) at P26. Animals were placed in a transparent cylinder (80mm diameter and 300mm height) and videotaped during at least 3 minutes. A minimum of 10 full weight-bearing rearings were recorded per animal, mice that did not meet this criterium within the timeframe were retested approximately 30 minutes later. Forepaw preference was calculated as ((non-impaired – impaired)/(non-impaired + impaired + both))x100%. All CRTs were videotaped and scored by researchers blinded to the experimental conditions.

***In vitro* models of dWMI**

Primary rat glial cultures

A mixed glial culture was acquired from P1-2 Sprague Dawley rat pup cortices, as described by Chen, Balasubramaniyan (5), with small changes. In summary, brains were isolated, cortices were dissected and the meninges were removed. Subsequently, cortices were pooled and minced, followed by dissociation using DNase I (10ug/l, Sigma Aldrich, D5025) and Trypsin (0.01%, Sigma Aldrich, T1426) solution in HBSS. Tissue was collected and dissociated by pipetting. The suspension was filtered through a 0.70µm filter twice. Cells were plated in poly-D-lysine-coated (0.1mg/ml, Sigma Aldrich, P6407) T75 culture flasks. For details on the media used, we kindly refer to the original paper by Chen, Balasubramaniyan (5). After a minimum of 10DIV, microglia and oligodendrocyte precursor cells (OPCs) were collected by mechanical detachment on an orbital shaker (see below for details). A second harvest was performed after a minimum of 7 addition DIV.

To mimic the *in vivo* inflammatory situation to induce maturation arrest in immature oligodendrocytes, microglia-conditioned medium (MCM) was produced. To detach microglia, culture flasks were shaken during 1 hour at 200rpm at 37°C. Culture medium containing the microglia was centrifuged (10 minutes, 1200rpm, RT) and microglia were counted. Microglia were plated at 0.5x106 cells per well in poly-L-ornithine (Sigma Aldrich, P3655)-coated 24 wells plates. At 24 hours after plating, culture medium was replaced by Basal Defined Medium (BDM) with- or without 50ng/ml LPS (Sigma, L4616) for 24 hours at 37°C. MCM-LPS and MCM+LPS was collected and pooled, filter sterilized (0.20µm) and stored at -80°C upon use.
After shaking to remove the microglia, culture flasks were refilled with fresh medium and shaken for an additional 20 hours at 200 rpm at 37°C to isolate OPCs. The cell suspension was collected and passed through a 20µm sterile screening pouch (Merck Millipore, NY2004700, Burlington, MS) to avoid potential contamination with microglia and astrocytes. OPCs were collected by centrifugation (100g during 10 minutes RT) and plated at 4.0x10E4 cells/well on poly-D,L-ornithine (Sigma Aldrich, P0421)-coated 24-wells plates in OPC medium (BDM with PDGF-aa (Peprotech, 100-13A) and bFGF (Peprotech, 100-18B)). OPC medium was changed every other day. For the OPC differentiation assay, see below at cocultures.

Primary mouse microglia culture

A primary microglia culture was prepared from P1 C57BL/6 mice cortices for cocultures. In short, cortices were dissected and meninges were removed. The tissue was minced and incubated with 0.25% trypsin (Sigma, T4799) in Gey's balanced salt solution (GBSS) containing 100 U/ml penicillin, 100 µg/ml streptomycin, and 30 mM D(+)-glucose during 15 minutes. The tissue suspension was dissociated by pipetting until homogenous and thereafter cultured in poly-L-ornithine-coated(Sigma, P3655) T75 flasks in DMEM/HamF10 (1:1) (Gibco, 41965-039 and 31550-023) supplemented with 10% FCS, 2mM

glutamine and antibiotics (see above). At DIV10-12 culture flasks were put on an orbital shaker during 20-22 hours (130-135rpm, 37°C) to detach microglia. Subsequently, microglia were collected by centrifugation (1200 rpm during 10 minutes RT), counted and seeded in poly-L-ornithine-coated 24-wells plates at a density of $1.5x10^5$ cells per well. Cocultures (see below) were started 24 hours later. After shaking the T75 flasks, new culture medium was added to each flask to allow a second microglia harvest after an additional 7-10 DIV.

Non-contact MSC-glia cocultures

To assess the effect of MSC modification on myelin production by OLs or activation of microglia, overexpressing-MSCs were embedded in gel inserts for a non-contact coculture between MSCs and primary OPCs or microglia respectively. In this coculture system, the effects of MSCs are mediated by their secretome, as MSCs in the gel insert are not able to have direct cell-contact with the primary cells in the lower well. Moreover, it allows both cell types to remain viable in their own culture medium. At 24 hours prior to the start of cocultures (and 24 hours after MSC transfection) $4.0x10^4$ modified MSCs (MSC-eGFP (control), MSC-IGF1, MSC-EGF, MSC-LIF, MSC-IL10 and MSC-IL11) were embedded in Hydromatrix gel (Sigma, A6982) transwell inserts (Merck Millipore, MCHT24H48) according to supplier's protocol.

For the OL differentiation experiment, OPC medium containing PDGF-AA and bFGF was replaced with either MCM+LPS or MCM-LPS when the majority of OLs displayed an immature pre-OL morphology (i.e. 4 days after OPC plating). Pro-differentiation factors: NAC (5µg/ml Sigma, A8199), CNTF (10ng/ml Peprotech, 450-50) and T3 (122ug/ml Sigma, T2752) were added to MCM+LPS or MCM-LPS to start differentiation of OPCs. Transwell inserts containing modified MSCs (MSC-eGFP (control, EV), MSC-IGF1, MSC-EGF, MSC-LIF, MSC-IL10 and MSC-IL11) or no MSCs as an empty insert control, were added to the wells directly or 24 hours (delayed) after induction of differentiation. Inserts were removed and OLs were fixated 72 hours after addition of MCM, with 4% PFA in PBS during 10 minutes.

At 24 hours after plating, co-cultures of MSCs and mouse microglia were started by adding 50ng/ml LPS (Sigma, L4515) to the microglia and putting transwell inserts containing modified MSCs (MSC-eGFP, MSC-IGF1, MSC-EGF, MSC-LIF, MSC-IL10 and MSC-IL11) to the wells. After 48 hours of coculture, the inserts were removed and the microglia supernatant was collected, aliquoted and stored at -80C° for ELISA.

ELISA

TNFα concentrations in the supernatant of microglia were measured using an ELISA kit for murine TNFα (Ucytech, Utrecht, The Netherlands) according to manufacturer's protocol. TNFα data of different experiments were normalized to positive control conditions (i.e. 50ng/ml LPS and empty insert without MSCs).

Luminex assay

The concentrations of 31 cytokines/chemokines in pooled microglia supernatant (n=3 per condition) were measured using a bioplex pro mouse chemokine assay (12009159, Biorad) according to the supplier's protocol. All concentrations were normalized to the EV-MSC condition (i.e. 50ng/ml LPS and an insert with EV-MSCs).

Immunocytochemistry of primary oligodendrocyte cultures

After fixation and washing in PBS, 2% BSA and 0.1% saponin in PBS was added to the wells to block nonspecific binding. Wells were incubated with primary antibodies (rabbit-anti-Olig2, AB9610. Merck Millipore; 1:1000, mouse-anti-MBP, SMI-94, Biolegend, 1:1000) overnight at 4°C. The following day, wells were washed with PBS, followed by incubation with alexafluor-594 and -488 conjugated secondary antibodies (Life technologies; 1:1000) for 1 hour at room temperature. Hoechst 33342 (Sigma) was used for nuclear counterstaining and wells were embedded in Fluorsave (Merck Millipore, 345789).

Six adjacent fields were imaged (10x), starting at a fixed distance of the well edges. The number of Olig2- and Hoechst- positive cells were counted using the analyze particles function in ImageJ v.1.47. The area of MBP+ staining was measured using manual thresholding analyses in ImageJ. In order to compare independent experiments, all results were normalized for the positive control (MCM+LPS; empty insert without MSCs).

Statistics

All data are shown as mean ± standard error of the mean (SEM). Statistics were performed using Graphpad Prism 8.3. Unpaired t-tests were used for comparison of two groups, or in case of unequal variances, non-parametric mann-whitney tests. For comparison of >2 groups, one-way ANOVA with Bonferroni posthoc tests were executed. In the event of unequal variances, a non-parametric Kruskal-Wallis test with Dunn's posthoc correction was used for comparison of multiple groups. p-values <0.05 were considered statistically significant. Sample sizes are mentioned in the figure captions.

Table S1: Overview of primer sequences used in validation qPCR

Symbol	Forward primer sequence	Reverse primer sequence
**Cxcl10**	*GCTGCCGTCATTTTCTGC*	*TCTCACTGGCCCGTCATC*
**Ccl3**	*CTGCCCTTGCTGTTCTTCTCTG*	*CGATGAATTGGCGTGGAATCTTC*
**IL1β**	*CAACCAACAAGTGATATTCTCCATG*	*GATCCACACTCTCCAGCTGCA*
**GAPDH**	*TGAAGCAGGCATCTGAGGG*	*CGAAGGTGGAAGAGTGGGAG*
**β-actin**	*AGAGGGAAATCGTGCGTGAC*	*CAATAGTGATGACCTGGCCGT*

Table S2: Validation chemokine expression changes

**RefSeq**	**Gene symbol**	**2^ΔCt**		**Fold Regulation validation**
		**SHAM D3**	**dWMI D3**	
**NM_021274**	Cxcl10	0,0000595	0,000362167	6,08
**NM_011337.2**	Ccl3	0,000143404	0,00037147	2,59

Table S3: Validation MSC secretome expression changes

**RefSeq**	**Gene symbol**	**2^ΔCt**		**Fold Regulation validation**
		**MSC D3**	**MSC D6**	
**NM_008361**	IL1β	0,000005414	0,0000152402	2,814970144
**NM_011337.2**	Ccl3	0,000015817	0,0000271900	1,719075577

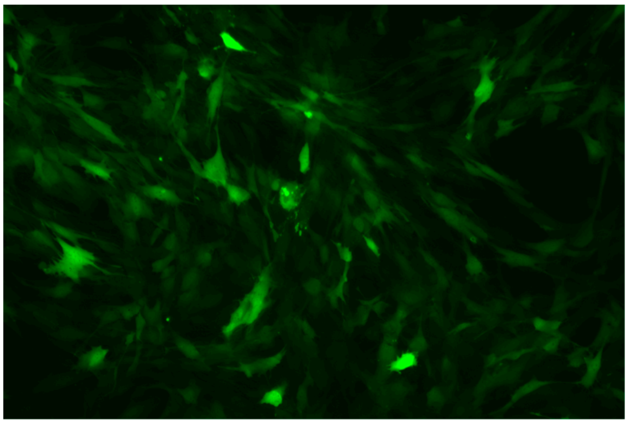

Figure S1. Representative fluorescent image (10x) of MSCs transfected with control adenoviral vector containing an eGFP transgene at a multiplicity of infection (MOI) of 4000 pfu/cell during 6 hours.

Table S4: Relative changes in cyto- and chemokine concentrations in the microglial supernatant measured using Luminex

	**Cxcl13**	**Ccl27**	**Cxcl5**	**Ccl11**	**Ccl24**	**Cx3Cl1**	**GMCSF**	**Ccl1**	**Ifnγ**	**IL1β**	**IL2**	**IL4**	**IL6**	**IL10**	**IL16**	**Cxcl10**
**LPS – MSC -**	0.57	0.49	**0.04**	0.66	**0.17**	**0.54**	**0.09**	0.66	0.56	0.31	0.45	0.56	**0**	**0.41**	0.77	**0.19**
**LPS + MSC -**	0.84	0,92	**0.06**	0.96	**0.76**	**0.76**	**0.26**	0.89	1.01	0.84	0.88	0.96	**0.30**	**0.94**	0.92	**0.96**
**LPS + EV-MSC**	1	1	**1**	1	**1**	**1**	**1**	1	1	1	1	1	**1**	**1**	1	**1**
**LPS + IGF1-MSC**	0.95	0.88	**0.98**	1	**0.30**	**0.96**	**0.84**	1	1	0.92	1.10	1	**0.74**	**1.04**	1.08	**1.03**
**LPS + EGF-MSC**	1.01	0.95	**1.48**	1.03	**0.68**	**1.34**	**1.96**	0.99	0.94	1.06	1.08	1.04	**1.90**	**0.99**	1.13	**2.20**
**LPS + LIF-MSC**	0.93	0.92	**1.29**	1.11	**0.98**	**1.15**	**1.53**	0.95	1.04	0.93	0.99	0.97	**1.26**	**0.91**	1.38	**2.35**
**LPS + IL11-MSC**	1.03	0.90	**2.90**	0.99	**0.70**	**1.28**	**1.98**	0.91	1.03	0.99	0.98	1.06	**1.44**	**1.02**	1.11	**1.19**
**LPS + IL10-MSC**	1.06	0.90	**3.11**	1.02	**0.69**	**1.29**	**1.69**	0.96	1.07	0.87	1.10	1.02	**1.12**	**13.81**	1.11	**1.22**
	**Cxcl11**	**Cxcl1**	**Ccl2**	**Ccl7**	**Ccl12**	**Ccl22**	**Ccl3**	**Ccl4**	**Ccl20**	**Ccl19**	**Ccl5**	**Cxcl16**	**Cxcl12**	**Ccl17**	**Tnfα**	
**LPS – MSC -**	0.48	**0.01**	**0.04**	**0.04**	**0.06**	0.57	0.05	0.16	0.47	0.46	0.01	**0.40**	0.75	0.75	**0.01**	
**LPS + MSC -**	0.94	**0.45**	**0.61**	**0.53**	**0.89**	0.94	1.05	1.03	0.94	0.93	1.21	**0.75**	0.92	0.97	**1.88**	
**LPS + EV-MSC**	1	**1**	**1**	**1**	**1**	1	1	1	1	1	1	**1**	1	1	**1**	
**LPS + IGF1-MSC**	1.04	**0.84**	**0.87**	**0.83**	**0.91**	1.05	1.03	1.03	1.22	1.03	0.98	**0.76**	0.96	1.01	**0.80**	
**LPS + EGF-MSC**	1.09	**1.42**	**1.19**	**1.18**	**0.86**	1.03	1.07	1.03	1.09	1	0.99	**0.92**	1.02	1.02	**0.72**	
**LPS + LIF-MSC**	1.01	**1.18**	**1.03**	**0.95**	**0.76**	1.28	0.99	0.97	1.08	0.94	1.07	**0.85**	1	1.04	**0.77**	
**LPS + IL11-MSC**	1.08	**1.60**	**1.34**	**1.37**	**0.99**	1.16	1.04	1.08	1.12	1	1.07	**0.73**	1.02	1.04	**0.83**	
**LPS + IL10-MSC**	1.06	**1.43**	**1.13**	**1.21**	**1.21**	0.94	0.91	0.91	1.31	0.99	0.89	**0.69**	1.01	1.04	**0.45**	

References Supplementary Information

1. Vaes JEG, van Kammen CM, Trayford C, van der Toorn A, Ruhwedel T, Benders MJNL, et al. Intranasal mesenchymal stem cell therapy to boost myelination after encephalopathy of prematurity. Glia. 2020;n/a(n/a).
2. van Tilborg E, van Kammen CM, de Theije CGM, van Meer MPA, Dijkhuizen RM, Nijboer CH. A quantitative method for microstructural analysis of myelinated axons in the injured rodent brain. Scientific reports. 2017;7(1):16492-.
3. Schneider CA, Rasband WS, Eliceiri KW. NIH Image to ImageJ: 25 years of image analysis. Nature Methods. 2012;9(7):671-5.
4. Zanier ER, Fumagalli S, Perego C, Pischiutta F, De Simoni M-G. Shape descriptors of the "never resting" microglia in three different acute brain injury models in mice. Intensive Care Med Exp. 2015;3(1):39-.
5. Chen Y, Balasubramaniyan V, Peng J, Hurlock EC, Tallquist M, Li J, et al. Isolation and culture of rat and mouse oligodendrocyte precursor cells. Nature protocols. 2007;2(5):1044-51.
